# Editorial: Revolutionizing sleep instability: advanced diagnosis and management of sleep disorders

**DOI:** 10.3389/frsle.2026.1835364

**Published:** 2026-05-25

**Authors:** Fábio Mendonça, Sheikh Mostafa, Antonio Ravelo-Garcia, Fernando Morgado-Dias

**Affiliations:** 1 Faculty of Exact Sciences and Engineering, University of Madeira, Funchal, Portugal; 2 Interactive Technologies Institute (ITI/LARSyS), Agência Regional para o Desenvolvimento da Investigação, Tecnologia e Inovação (ARDITI), Funchal, Portugal; 3 Universidad de Las Palmas de Gran Canaria, Institute for Technological Development and Innovation in Communications, Las Palmas de Gran Canaria, Spain

**Keywords:** artificial intelligence, case report, device validation, sleep instability, wearable devices

Sleep instability is a common factor across a wide range of sleep disorders, yet its assessment remains fragmented. Conventional clinical practice often relies on single summary measures, such as the apnea–hypopnea index or subjective symptom scales, which do not fully capture the complexity of sleep physiology or patient experience. At the same time, advances in signal processing, artificial intelligence, and wearable technology are changing how sleep can be measured and interpreted. This Research Topic brings together contributions that reflect this transition, combining clinical, technological, and mechanistic perspectives to better define how sleep instability can be assessed and managed.

The articles included in this collection highlight a shift from single-metric evaluation toward multidimensional characterization of sleep. Across different study designs, including original research, technical validation, and case reports, the contributions show that sleep disorders cannot be fully understood through isolated indices. Instead, they require integration of physiological signals, symptom profiles, and context-specific clinical information. Together, the studies provide a coherent view of how emerging tools and approaches can complement established methods.

One contribution (Amiri et al.) examines inconsistencies between subjective and physiological measures in obstructive sleep apnea. In a retrospective analysis, a substantial proportion of patients showed disagreement between Epworth Sleepiness Scale scores and apnea–hypopnea index values, with some patients reporting low sleepiness despite severe apnea and others reporting high sleepiness with only mild disease. These findings illustrate that symptom perception and physiological burden do not always align. They also support caution when relying on a single metric to define disease severity and suggest that combined assessment approaches are necessary.

Another study (Saeb et al.) evaluates the performance of a wrist-worn wearable device against polysomnography for sleep measurement. The device showed high sensitivity for detecting sleep vs. wake and moderate agreement for sleep staging, with some systematic differences in estimated sleep parameters. This work highlights the potential and the current limitations of wearable technologies. While such devices can support longitudinal monitoring in real-world settings, validation against reference standards remains necessary, especially for detailed sleep architecture.

A complementary perspective is provided by a study on AI-driven sleep apnea screening using overnight blood oxygen saturation signals (Hoang and Liang). This work reviews current approaches that rely on SpO_2_ data to detect sleep apnea and discusses how machine learning methods can extract meaningful patterns from relatively simple physiological signals. These approaches, which focus on oximetry, can provide a simpler, more accessible alternative to full polysomnography while still capturing relevant information about respiratory events and oxygen desaturation. The study highlights the potential of these methods for large-scale screening and their current limitations, particularly regarding generalization and clinical validation.

Beyond measurement, the collection also addresses sleep as a dynamic outcome that can change with treatment and clinical context (Ren et al.). Real-world data on sleep efficiency in hospitalized patients show that sleep parameters are responsive to short-term clinical interventions and influenced by multiple factors. This perspective emphasizes that sleep metrics are more than diagnostic tools, as they can also be markers of treatment response and recovery.

The case-based contributions extend the scope of the Topic by focusing on less typical manifestations of sleep instability. One report (Mulas et al.) describes persistent REM sleep without atonia in an adult with pediatric acute-onset neuropsychiatric syndrome, suggesting that sleep abnormalities may reflect underlying neuroimmune mechanisms and altered REM regulation. Another case study (Marsh) presents the use of hypnotherapy for chronic somniloquy, with reported improvement after several sessions, indicating that behavioral and non-pharmacological interventions may have a role in selected parasomnias. These reports show that individualized assessment remains important, particularly in conditions where standard diagnostic frameworks may not fully apply.

Taken together, the contributions in this Research Topic point toward a more integrated approach to sleep medicine. There is a clear movement away from reliance on single indices toward the use of multimodal data, including physiological signals, wearable-derived measures, artificial intelligence-based methods for data analysis and interpretation, and clinical observations. At the same time, the studies show that new technologies should complement, rather than replace, established methods such as polysomnography. Careful validation, interpretation, and clinical integration remain essential.

Future work could, therefore, focus on combining different sources of information into coherent models of sleep instability that are clinically meaningful and scalable. This includes improving the interpretability of artificial intelligence-based methods, ensuring that wearable technologies perform reliably across populations, and linking sleep metrics to patient-centered outcomes. In parallel, case-based and mechanistic studies will continue to play a role in identifying less common presentations and refining our understanding of underlying pathways.

